# High Precision Antibody-Free Microtubule Labeling for Expansion Microscopy

**DOI:** 10.21769/BioProtoc.5539

**Published:** 2025-12-20

**Authors:** Rajdeep Chowdhury, Donatus Krah, Antonios Ntolkeras, Alina Heimbrodt, Ali H. Shaib

**Affiliations:** 1 Department of Neuro- and Sensory Physiology, University Medical Center Göttingen, Göttingen, Germany; 2 Department of Chemistry, GITAM School of Science, GITAM, Hyderabad, Telangana, India

**Keywords:** Detergent-extraction, NHS-ester labeling, Microtubules, Expansion microscopy, High-precision imaging

## Abstract

Expansion microscopy (ExM) enables nanoscale imaging of biological structures using standard fluorescence microscopes. Accurate labeling of cytoskeletal filaments, such as microtubules, remains challenging due to structural distortion and labeling inaccuracy during sample preparation. This protocol describes an optimized method combining detergent extraction and NHS-ester labeling for high-precision visualization of microtubules in expanded samples. Cytoplasmic components and membranes are selectively removed, preserving the ultrastructure of the microtubule network. Microtubules are digested into peptides during expansion and subsequently labeled at their N-termini using NHS-ester dyes, eliminating the need for antibodies. Effective fluorophore displacement of ~1 nm or lower is achieved, depending on the applied expansion factor. The protocol is compatible with both in vitro and cellular samples and can be integrated into a wide range of ExM workflows. Labeled microtubules can serve as internal reference standards for correcting expansion factors in ExM datasets.

Key features

• Employs detergent extraction with accessible commercial reagents to isolate cytoskeletal structures and reduce background from membranes and cytoplasmic proteins in fluorescence microscopy.

• Avoiding excessive aldehyde fixation preserves amines required for gel polymer integration while NHS-ester labeling of tubulin amines reduces linkage error, enabling accurate molecular localization.

• Compatible with post-expansion workflows; labels newly generated peptide N-termini after digestion, enabling high-resolution fluorescence imaging with minimal linkage error and high signal-to-noise ratio (SNR).

• Suitable for integration into diverse ExM protocols and useful as a reference standard for expansion factor correction.

## Graphical overview


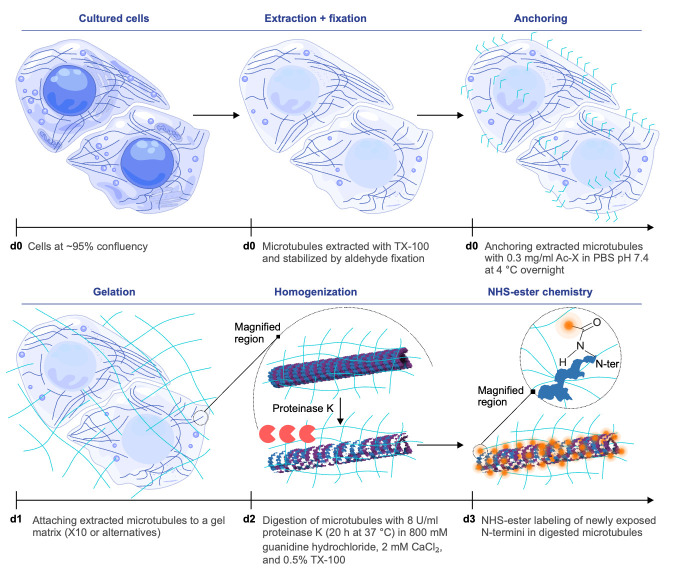


**Antibody-free strategy for homogeneous expansion and direct microtubule labeling.** In this schematic, “d” followed by a number indicates the day on which each step is performed. Microtubules are extracted and chemically stabilized, then covalently anchored to the expandable gel matrix. During the homogenization step on d2, a magnified region is shown to illustrate the process at the single microtubule level, representative of the overall sample. Proteolysis ensures uniform softening of the specimen and exposes new protein N-termini, which can be directly tagged by NHS-esters. The samples are subsequently expanded by water infusion, which swells the gel isotropically. This approach minimizes linkage error, since the effective contribution of the dye size (~1 nm) becomes negligible when scaled down by the expansion factor. The combination of homogeneous anchoring, complete digestion, direct chemical labeling after partial expansion, and isotropic expansion enables dense, antibody-free visualization of microtubules at nanoscale resolution.

## Background

The advent of super-resolution microscopy has transformed cell biology by overcoming the diffraction limit and enabling visualization of subcellular structures with nanometer-scale precision [1–3]. Expansion microscopy (ExM [4]) achieves this not through complex optics but by physically enlarging specimens embedded in swellable hydrogels. The final resolution depends on the achieved expansion factor (e.g., 4× [4,5], 10× [6], 20× [7], or higher with gel iteration protocols [8–10]), which is determined by the composition of the hydrogel and the extent of protein homogenization [4–10]. Accurate calibration of the expansion factors requires internal reference structures with well-defined geometry and consistent labeling, which remains a significant challenge. For a considerable time, the nuclear pore complex (NPC) was used as a reference structure in both super-resolution [11,12] and expansion microscopy [9,13–15], owing to its well-defined geometry. Yet, with the advancement of ExM protocols and their integration with super-resolution methods, NPCs became less practical for this role. Their relatively large diameter means that they no longer provide a stringent measure of the effective resolution or expansion factor achievable today. We therefore turned to a smaller and more uniform intracellular structure: the microtubule network [15]. Microtubules are particularly well suited for this purpose because their cylindrical architecture, assembled from regularly arranged α/β-tubulin polymers with an outer diameter of about 25 nm, is highly predictable and conserved [16,17]. Their widespread presence in cells and uniform shape essentially make them a good choice for nanoscale rulers [15,18]. However, imaging microtubules in cells typically relies on antibody-based labeling [19], which introduces a spatial displacement of 30–80 nm between the fluorophore and the tubulin backbone due to the physical size and orientation of the antibody layers [15,19]. Nanobodies can reduce this displacement because of their smaller size, although inconsistencies in epitope accessibility still introduce variability.

To address this limitation, we developed a protocol that combines detergent extraction and NHS-ester labeling, bypassing the need for immunostaining. Cells are treated with Triton X-100 to remove membranes and soluble cytoplasmic proteins, preserving the cytoskeletal framework. Fluorescent NHS-esters are then used to label accessible amine groups, allowing direct tagging of the protein backbone with a displacement of approximately 1 nm or lower, depending on the expansion factor. Importantly, labeling targets not only lysine side chains and N-termini of intact proteins but also newly exposed amines generated during the digestion process. This significantly increases the density of labeling and enhances the signal-to-noise ratio. Microtubules, being the most prominent retained filaments, are readily distinguished from smaller cytoskeletal structures such as actin filaments. Their diameters are approximately 2–3 times larger than those of other cytoskeletal components, making them particularly amenable to visualization using our high-precision expansion protocol. This methodological advantage is consistent with previous findings [20], which effectively visualized and spatially resolved microtubules, intermediate filaments, and actin filaments within the same fixed cells. The digestion buffer contained 2 mM Ca^2+^, which may induce minor perturbations in actin organization. However, because the cells were chemically fixed prior to digestion, the overall integrity of the actin cytoskeleton is expected to remain largely preserved. The protocol has been validated using high-resolution techniques, including one-step nanoscale expansion (ONE) microscopy and expansion stimulated emission depletion (ExSTED) [15], with achieved resolutions of under 5 nm and approximately 8 nm, respectively. It is compatible with a variety of ExM workflows, including standard 4× [4] and 10× [6] protocols as well as U-ExM [5]. By eliminating antibody-induced displacement and providing a reliable cytoskeletal reference, this method improves the accuracy of structural analyses in ExM, including the study of microtubule-associated proteins and post-translational modifications in their native context.

**Critical steps of the protocol**

This method builds on an established cytoskeleton isolation protocol [21], incorporating key optimizations for ExM compatibility: mild fixation (0.5%–1% glutaraldehyde), controlled extraction using 2% Triton X-100 with Taxol, and post-expansion NHS-ester labeling. Each of these steps is outlined and discussed in detail in Table 1.

**Table 1.** Critical concepts for reliable microtubule expansion and visualization

Critical step	Description
Mild fixation	Uses low glutaraldehyde concentrations to preserve microtubule architecture while avoiding excessive cross-linking. This balance ensures efficient gel anchoring and prevents artifacts from rigid or incompletely digested structures, which in turn can lead to non-homogeneous expansion factors.
Controlled extraction	Employs 2% Triton X-100 with Taxol to solubilize membranes without depolymerizing microtubules. This maintains cytoskeletal integrity and ensures that filaments remain accessible for anchoring and subsequent expansion.
Post-expansion labeling	Applies NHS-ester dyes after homogenization, when peptides are uniformly accessible. This minimizes labeling bias, reduces linkage error compared to antibodies, and produces dense, isotropic signal along microtubules. Fluorescein is suited for confocal imaging or ONE microscopy, whereas STAR635P enables successful STED imaging on expanded specimens. Other NHS-ester dyes can be used, but hydrophobic dyes should be avoided as they aggregate on negatively charged peptides and generate nonspecific bright signal.

## Materials and reagents

**Cell line**

1. HeLa cells were used in this protocol for microtubule extraction and subsequent labeling using NHS-ester chemistry

**Reagents**

1. Dulbecco’s modified Eagle’s medium (DMEM) (Gibco, catalog number: 41965039)

2. Trypsin-EDTA (0.25%), phenol red (Gibco, catalog number: 25200072)

3. Fetal bovine serum (FBS) (Gibco, catalog number: 10270106)

4. Penicillin-streptomycin (Thermo Fisher Scientific, catalog number: 15140148)

5. Dulbecco’s phosphate-buffered saline (PBS) (1×) (Gibco, catalog number: 14190169)

6. Piperazine-(N,N’-bis(2-ethanesulfonic acid) (PIPES) sodium salt (Merck, catalog number: P2949)

7. Tris(hydroxymethyl)aminomethane (Tris) base (Merck, catalog number: 252859)

8. Magnesium chloride (MgCl_2_) (Merck, catalog number: 208337)

9. Ethylene glycol-bis(2-aminoethylether)-N,N,N′,N′-tetraacetic acid (EGTA) (Merck, catalog number: E3889)

10. Triton X-100 (Merck, catalog number: T8787)

11. Polyethylene glycol (PEG) (MW 20,000) (Merck, catalog number: 86101)

12. Phalloidin (Thermo Fisher Scientific, catalog number: P3457)

13. Taxol (paclitaxel) (Merck, catalog number: T7402)

14. Electron microscopy–grade glutaraldehyde solution (50%) (Merck, catalog number: G7651)

15. Acryloyl-X SE (succinimidyl ester) (Thermo Fisher Scientific, catalog number: A20770)

16. Fluorescein NHS ester (Thermo Fisher Scientific, catalog number: 46409)

17. Anhydrous dimethyl sulfoxide (DMSO) (Merck, catalog number: 276855)

18. Sodium bicarbonate (Merck, catalog number: S6014)

19. Guanidine hydrochloride (Merck, catalog number: G3272)

20. Calcium chloride (CaCl_2_) (Merck, catalog number: C4901)

21. Proteinase K (Merck, catalog number: P4850)

**Solutions**

1. PIPES stock solution (see Recipes)

2. MgCl_2_ stock solution (see Recipes)

3. CaCl_2_ stock solution (see Recipes)

4. EGTA stock solution (see Recipes)

5. PEM buffer solution (see Recipes)

6. Taxol stock solution (see Recipes)

7. Phalloidin stock solution (see Recipes)

8. Extraction solution (see Recipes)

9. Fixation solution (see Recipes)

10. Acryloyl-X SE stock solution (see Recipes)

11. Digestion buffer (see Recipes)

12. Digestion solution (see Recipes)

13. Sodium bicarbonate buffer solution (see Recipes)

14. Fluorescein NHS ester stock solution (0.2 g/mL) (see Recipes)

**Recipes**

**1. PIPES stock solution (200 mM in 500 mL)**

Dissolve 32.43 g of PIPES sodium salt in 450 mL of ddH_2_O, add 1 M KOH until it dissolves, add ddH_2_O to make up the volume to 500 mL, and adjust the pH to 6.9. Store at 4 °C for short-term and -20 °C for long-term storage.

**2. MgCl_2_ stock solution (10 mM in 500 mL)**

Dissolve 0.476 g of MgCl_2_ powder in 500 mL of ddH_2_O. Store at 4 °C for short-term and -20 °C for long-term storage.

**3. CaCl_2_ stock solution (500 mM in 20 mL)**

Dissolve 1.1 g of CaCl_2_ powder in 20 mL of ddH_2_O. Store in a 50 mL centrifuge tube at 4 °C.

**4. EGTA stock solution (10 mM in 200 mL)**

Dissolve 0.76 g of powder in 200 mL of ddH_2_O. Store at 4 °C for short-term and -20 °C for long-term storage.

**5. PEM buffer solution (100 mM in 500 mL)**

Mix 250 mL of PIPES with 50 mL of MgCl_2_ stock and 50 mL of EGTA stock. Adjust the remaining 150 mL volume with ddH_2_O. Check the pH and adjust it by gradual addition of 1 M KOH using a micropipette while stirring continuously until the solution reaches the target pH of 6.9. Final composition of the PEM buffer becomes [PIPES] = 100 mM, [MgCl_2_] = 1 mM, and [EGTA] = 1 mM. This solution can be stored at room temperature (RT).

**6. Taxol stock solution (1 mM)**

Dissolve 1 mg of Taxol powder in 1.17 mL of anhydrous DMSO and store it at -20 °C. It is recommended to make small aliquots of 10 μL for single use and not to reuse the same aliquot.

**7. Phalloidin stock solution (1 mM)**

Dissolve 1 mg of phalloidin powder in 1.25 mL of anhydrous DMSO to get a 1 mM stock solution of phalloidin. It should be stored at -20 °C and may be reused.

**8. Extraction solution (50 mL)**

**Table:** 

Reagent	Final concentration	Volume/weight
Triton X-100	2% (v/v)	1 mL
PEG	4% (w/v)	2 g
Taxol stock solution (1 mM)	2 μM	100 μL
Phalloidin stock solution (1 mM)	2 μM	100 μL
PEM buffer	~100 mM	48.8 mL

Dissolve 2 g of PEG 20000, 1 mL of Triton X-100, 100 μL of Taxol stock solution (1 mM), and 100 μL of phalloidin stock solution (1 mM) in 48.8 mL of PEM buffer. Phalloidin is included in the extraction solution to stabilize filamentous actin (F-actin) and prevent its depolymerization during detergent extraction. It binds specifically and with very high affinity to only polymerized F-actin, thereby preserving existing actin filaments without influencing microtubule integrity. This selective binding stabilizes actin structures against chemical or mechanical disruption while allowing simultaneous maintenance of microtubules, which are independently stabilized by Taxol. Thus, the inclusion of phalloidin helps retain the native architecture of both filament systems for accurate structural analysis.

**9. Fixation solution**

Dilute the EM-grade glutaraldehyde to 0.5% in 100 mM PEM buffer (to pH 7.3). Glutaraldehyde is chemically unstable in aqueous solution and tends to undergo spontaneous polymerization and oxidation over time, especially near neutral pH. These reactions reduce the proportion of active monomeric aldehyde groups responsible for effective protein cross-linking and compromise fixation efficiency. Consequently, freshly diluted EM-grade glutaraldehyde ensures maximal reactive aldehyde availability and reproducible fixation of cellular structures. EM fixation guidelines recommend preparing glutaraldehyde solutions immediately before use to maintain optimal fixative performance. Hence, we recommend using a freshly prepared solution.

**10. Acryloyl-X SE stock solution (10 mg/mL)**

Dissolve 5 mg of material in 500 μL of anhydrous DMSO to get a 10 mg/mL stock solution and store it at -20 °C.

**11. Digestion buffer (100 mL)**

**Table:** 

Reagent	Final concentration	Volume/weight
Tris base	100 mM	1.2 g
CaCl_2_ stock solution (500 mM)	2 mM	400 μL
Triton X-100	0.5%	500 μL
Guanidine hydrochloride	800 mM	7.6 g
ddH_2_O	N/A	99 mL

Dissolve 1.2 g of Tris base in 99 mL of ddH_2_O, add 400 μL of 500 mM CaCl_2_ stock solution to it, and adjust the pH to 8. Then, add 500 μL of Triton X-100 and 7.6 g of guanidine hydrochloride to it and mix using a magnetic stirrer until a clear solution is obtained. Remember not to add Triton X-100 and guanidine hydrochloride before adjusting the pH as they can damage the electrode. The solution can be stored at -20 °C for 6 months. We do not recommend freeze-thawing this solution multiple times; hence, it is better to make 1.5 mL aliquots in microcentrifuge tubes.

**12. Digestion solution**

The digestion buffer was thawed from frozen stocks and incubated at 37 °C for 30 min prior to use. Proteinase K (800 U/mL) was diluted 1:100 in the buffer, yielding a working concentration of 8 U/mL. For sample processing, 2 mL of the digestion solution was used per well of a 6-well plate, or 600 μL per well of a 12-well plate, ensuring complete immersion of the gels.

**13. Sodium bicarbonate buffer solution (100 mM at pH 8)**

Dissolve 4.2 g of sodium bicarbonate in 500 mL of ddH_2_O, adjust the pH to 8.0, and store the solution at RT.

**14. Fluorescein NHS ester stock solution (0.2 g/mL)**

Dissolve 1 g in 5 mL of anhydrous DMSO to obtain a 200 mg/mL solution. Preserving DMSO in an anhydrous state is challenging as it is highly hygroscopic and readily absorbs moisture from the atmosphere, which can compromise its anhydrous nature and affect solubility for moisture-sensitive reagents such as NHS esters. Therefore, DMSO should be stored in a tightly sealed amber glass bottle under dry conditions at RT (15–30 °C) and protected from light to prevent moisture uptake. NHS-ester dyes are inherently sensitive to hydrolysis, particularly in the presence of moisture. Once dissolved in anhydrous DMSO, their stability decreases markedly, and such stock solutions should be used within three months to prevent hydrolysis and loss of coupling efficiency. We recommend preparing small aliquots of 50–100 μL (for single use) and storing them at -20 °C.

**Laboratory supplies**

1. 12-well cell culture plate (Thermo Fisher Scientific, catalog number: 150200)

2. 6-well cell culture plate (Thermo Fisher Scientific, catalog number: 140675)

4. 25 cm^2^ tissue culture flask (Thermo Fisher Scientific, catalog number: 156367)

5. Microscope slides (Epredia, catalog number: J1800AMNZ)

6. 22 × 40 mm #1.5 coverslip (Electron Microscopy Sciences, catalog number: 72204-03)

7. 60 mm Petri dish (Fisher Scientific, catalog number: FB0875713A)

8. Expansion chamber (Corning, catalog number: 431111)

9. Polystyrene weighing tray (Merck, catalog number: HS1421C)

## Equipment

1. Cell culture incubator (Labotec, model: Incubator C200)

2. Double-distilled water dispenser (Sartorius, model: Arium^®^ Pro)

3. -80 °C freezer

4. -20 °C freezer

5. Refrigerator (4 °C)

6. Orbital shaker (Heidolph, model: Rotamax 120)

7. Phase contrast microscope (Zeiss, model: Axiovert 25)

8. Fluorescence microscope (Olympus, model: IX71)

9. STED microscope (Leica Microsystems, model: SP5)

10. 3D STED microscope (Leica Microsystems, model: Stellaris 8)

11. Abbe-refractometer (Krüss Optronic GmbH, Germany, model: AR4)

## Software and datasets

1. ImageJ version 1.53j was used. ONE plugin is compatible with newer versions.

2. CorelDraw

## Procedure

**A. Cell culture**

1. Culture HeLa cells in a tissue culture flask using DMEM media supplemented with 10% (v/v) FBS and 100 U/mL penicillin-streptomycin in 5% (v/v) CO_2_-enriched air at 37 °C.

2. For proper culture maintenance, grow cells to ~95% confluency and split using Trypsin-EDTA.

a. Estimate the extent of confluency by examining the coverage of the culture surface and assessing intracellular spaces qualitatively.

b. For detergent extraction, split cells the night before and place them onto round coverslips (15 mm diameter) inside a 12-well cell culture plate. Allow them to grow overnight in the same media.

c. Optimize the number of cells in such a way that they reach ~95% confluency overnight.

**B. Detergent extraction**

1. Upon reaching 95% confluency, carefully aspirate the culture medium to avoid disturbing the adherent cell monolayer.

2. Gently wash the cells with prewarmed PBS (37 °C) to remove residual serum proteins and metabolic byproducts, ensuring a clean baseline for subsequent extraction.

3. Replace PBS with a microtubule (MT)-stabilizing extraction buffer and incubate for precisely 7 min at 37 °C.

a. Prepare the MT-stabilizing buffer using PEM buffer (100 mM PIPES, 1 mM EGTA, 1 mM MgCl_2_, pH 6.9) supplemented with 2% (v/v) Triton X-100, 4% (w/v) PEG, 2 μM Taxol, and 2 μM phalloidin.

*Note: This buffer composition facilitates selective permeabilization of the plasma membrane while maintaining the polymerized state of microtubules. Taxol and PEM buffer together stabilize tubulin polymers by suppressing depolymerization, PEG provides osmotic support and minimizes structural collapse, and phalloidin aids in preserving actin filaments to maintain cytoskeletal architecture during extraction.*

4. To halt extraction and remove residual detergent, wash the cells three times with PEM buffer at RT. This helps maintain cytoskeletal stability by providing optimal pH while chelating free calcium, which reduces the activity of released lysosomal enzymes.

5. Fix the cells with a freshly prepared fixation solution for 20 min at RT, followed by three washes with PBS at pH 7.4 to remove the excess fixatives.

6. To assess the quality of detergent extraction prior to initiating the expansion protocol, wash extracted cells three times with sodium bicarbonate buffer, for 5 min each.

7. Perform fluorescent labeling using NHS-ester chemistry:

a. Prepare a 1,000-fold dilution of fluorescein NHS-ester (0.2 mg/mL stock) in bicarbonate buffer.

b. Incubate the cells at room temperature in the above solution for 30 min.

c. Remove excess, unreacted dye by three washes in PBS with gentle shaking (10 min each).

d. Mount the coverslips on a glass slide using 5 μL of water and subsequently image them by fluorescence microscopy to evaluate extraction efficiency.

**Figure 1.**
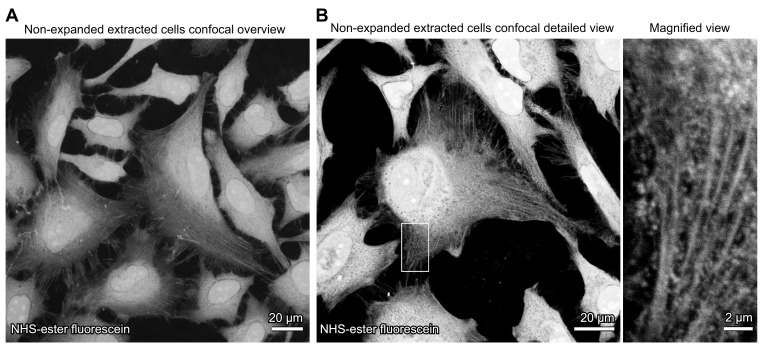
Visualization of detergent-extracted cells by NHS-ester labeling in non-expanded cells. Confocal images of non-expanded detergent-extracted cells directly labeled with NHS-ester fluorescein, marking bulk protein structures. (A) Overview image reveals the overall cellular confluency. (B) A detailed view followed by a higher magnification region highlights preserved cytoskeletal and proteinaceous structures following detergent extraction.

*Note: Representative images of non-expanded extracted cells are shown in Figure 1.*

**C. Anchoring for ExM**

*Note: We used NHS-ester chemistry to anchor proteins into the gels, which primarily attacks lysines on the tubulins.*

1. Incubate the coverslips overnight at 4 °C with 0.3 mg/mL acryloyl-X SE (3% of the stock solution) in PBS (pH 7.4).

2. Next morning, wash the coverslips 3× with PBS buffer.

**D. Gelation and homogenization**

**Figure 2.**
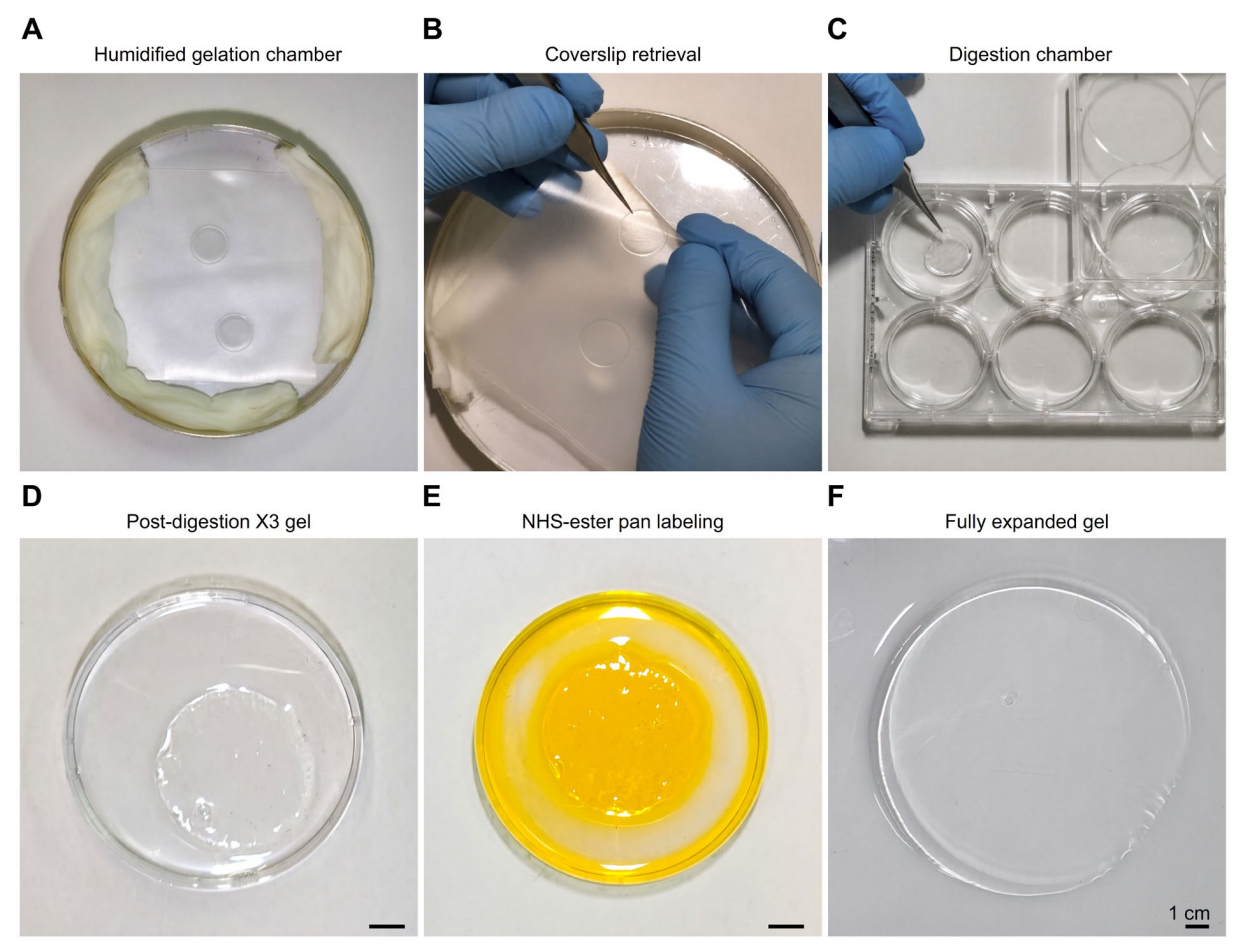
Overview of the X10 expansion protocol, from gel preparation to labeling and final expansion. (A) A gelation chamber was prepared from a round Petri dish; the outer surface was wrapped in aluminum foil to minimize photodamage, and the interior was lined with water-soaked tissue paper to maintain humidity and prevent dehydration at the gel edges. (B) The gel was removed by first taking out the parafilm from the chamber, then gently separating the gel from the parafilm, and finally detaching the coverslip with tweezers. (C) The gel attached to the coverslip was transferred to a 6-well plate and incubated with 10 mL of digestion solution. (D) After digestion, the gel was transferred to a Petri dish, washed three times with bicarbonate buffer, and incubated with fluorescein solution for 30 min under gentle shaking. (E) The gel after partial expansion and labeling with NHS-ester fluorescein is shown, followed by three washes with bicarbonate buffer to remove unbound dye. (F) A fully expanded gel after six rounds of ddH_2_O exchange is displayed, with optimal results obtained by leaving the gel in water overnight before use. Images were acquired with a Samsung Galaxy S25 Ultra smartphone, and angle distortion and lighting were corrected using Adobe Lightroom v8.4, 2025.

*Note: This protocol describes the preparation of samples for high-precision expansion microscopy. Expansion factors ranging from 4× to 20× were obtained by modifying the polymer composition and oxygen purging conditions [2,4,8]. All experiments in this study were performed using the X10 gel recipe [6,18], although the extracted microtubules are in principle compatible with other gel formulations. The ultrastructure expansion microscopy (U-ExM) protocol [5] is also compatible with this preparation.* ***Figure 2*** *provides a visual guide for researchers to reproduce the X10 expansion protocol. Steps involved in gelation and homogenization are given below.*

1. Pipette out 80 μL of gel monomer solution onto parafilm and overlay it with inverted coverslips containing extracted microtubules. Perform the gelation in a custom-designed humidified chamber to prevent drying at the edges, ensuring uniform polymerization and consistent final expansion (**Figure 2A**).

2. Incubate the chamber at RT for 12 h.

3. After polymerization, carefully detach the gels from the parafilm and transfer to a 6-well plate (**Figures 2B, C**).

*Note: This step is technically sensitive, as incomplete detachment can result in tearing of the gel or sample loss.*

4. Apply 1,000 μL of digestion solution to the gel, and homogenize it by incubation for 20 h at 37 °C. At this stage, the gel expands approximately threefold (**Figure 2D**).

*Note: Enzyme-assisted homogenization represents a relatively strong disruption method compared to heat-based lysis [6,18,22,23], leading to more extensive peptide bond cleavage and a higher number of free amino termini for NHS-ester labeling, which in turn enhances labeling density and signal-to-noise ratio. While enzyme-assisted homogenization was used here, alternative methods such as heat-based disruption may also be applied.*

**E. NHS-ester labeling**

1. Post-digestion, transfer the gel to a 60 mm Petri dish and wash three times with 100 mM bicarbonate buffer.

2. At the final wash, dilute 10 μL of fluorescein NHS-ester (or an alternative dye, at a concentration of 0.2 mg/mL) stock solution into 10 mL of bicarbonate buffer by vigorous shaking and add the mixture immediately to the Petri dish.

3. Incubate the gel in this solution for 30 min on a gentle shaker at low speed, ensuring it remains fully immersed.

*Note: A representative gel stained with fluorescein NHS-ester is shown in* ***Figure 2E***. *Staining at this intermediate stage, after digestion and prior to full expansion, allows efficient dye penetration while markedly reducing the amount of fluorescent dye needed.*

**F. Expansion of the gel**

1. Transfer the gel to a specially designed expansion chamber and wash at least 6 times with ddH_2_O until fully expanded (~15 cm diameter).

*Note:* ***Figure 2F*** *provides a visual of the final, fully expanded gel, confirming the successful X10 expansion achieved through the protocol. Detailed troubleshooting of the expansion procedures is available in [24]. The refractive index of the gel–glass solution was determined by placing a small gel slice onto the prism of the refractometer and reading the value at 20 °C according to the instrument's calibration, which yielded the value of 1.33331 as previously described [15].*

**Figure 3.**
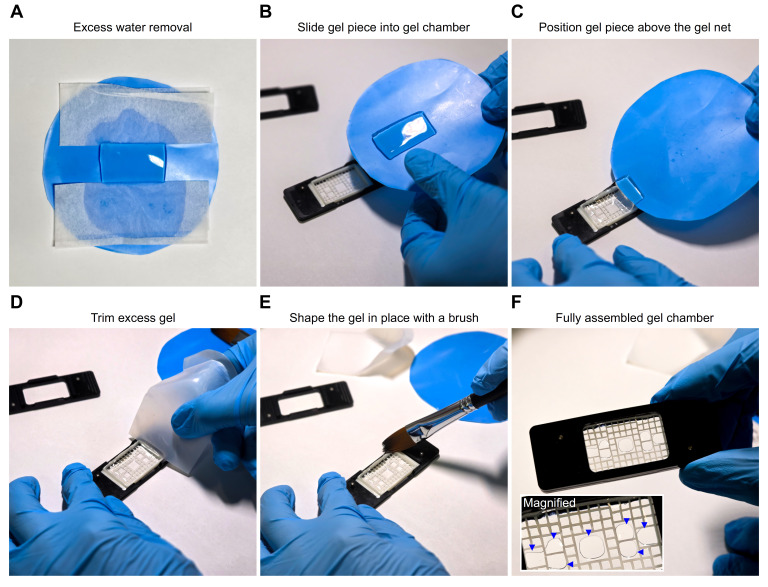
Assembly of the gel chamber for stable mounting of expanded samples during high-resolution imaging. (A) A rectangular gel piece was placed on a flat weighing tray (blue), and excess water was removed from two opposite sides using a nitrocellulose membrane. (B) The appearance of the gel after blotting excess water is shown. (C) The gel was carefully positioned on the supporting net by gradually sliding it into place. (D) The gel was cut slightly larger than the net, as recommended to ensure full coverage, and the excess was trimmed with a piece of the weighing tray to achieve a precise fit. (E) Excess gel fragments were then removed from the chamber using a paintbrush. (F) Finally, the top lid was placed onto the bottom frame, securing the net and gel to provide stability for high-resolution imaging. The blue arrows shown in the magnified region indicate air-free contact areas between the gel and the coverslip, which define the regions suitable for imaging. Images were acquired with a Samsung Galaxy S25 Ultra smartphone, and angle distortion and lighting were corrected using Adobe Lightroom v8.4, 2025.

**G. Sample preparation and mounting**

1. Construct a customized chamber to hold gel slices during imaging; the chamber consists of an aluminum holder designed to fit a 22 × 40 mm #1.5 coverslip.

*Note: The chamber design is described in detail in Shaib et al., 2024, Supplementary Figure 7 [18].*

a. To stabilize the coverslip, apply a thin layer of grease (e.g., Vaseline) around the edges of the holder.

b. Place a supporting net on top of the coverslip.

*Note: This net is a critical component, as it enables the gel and net to be removed together from the chamber to eliminate excess water that may accumulate between the coverslip and the net.*

c. Close the chamber with a top lid that secures the net firmly in place.

2. Gel mounting: For visual guidance, **Figure 3** illustrates the key steps involved in preparing and mounting expanded samples for imaging.

a. Gently blot the excess water from the gel before mounting (**Figure 3A, B**).

*Note: This step balances two requirements: removing enough water to prevent gel flotation and drift during imaging, while retaining sufficient moisture to avoid drying and deformation.*

b. Determine the orientation of the gel. For cell samples, the embedded structures are often visible as surface irregularities, whereas other preparations may require microscopic inspection to identify the correct side.

c. Flip the gel if the sample is located on the top surface so that the sample faces the coverslip. To achieve this, place a second flat piece of the weighing tray on top of the gel and invert the stack in a single motion.

d. Once properly oriented, transfer the gel carefully onto the net (**Figure 3C**).

e. Trim the edges with a piece of weighing tray to achieve a precise fit (**Figure 3D, E**).

*Note: This step is essential for minimizing sample drift during imaging.*

f. Finally, secure the lid of the custom chamber lid and press the gel and net against the bottom frame to ensure stable positioning throughout high-resolution acquisition (**Figure 3F**).

**H. Fluorescence imaging**

In principle, the user can choose any suitable fluorescence microscope to image non-expanded and expanded microtubule samples.

Here, we provide the details of the imaging methodology that we employed. For initial assessments of microtubule morphology, we used an Olympus IX71 epifluorescence microscope equipped with a 100×, 1.41 NA oil immersion objective. Images were acquired with an F-view II CCD camera, corresponding to a pixel size of 64.5 nm.

**Figure 4.**
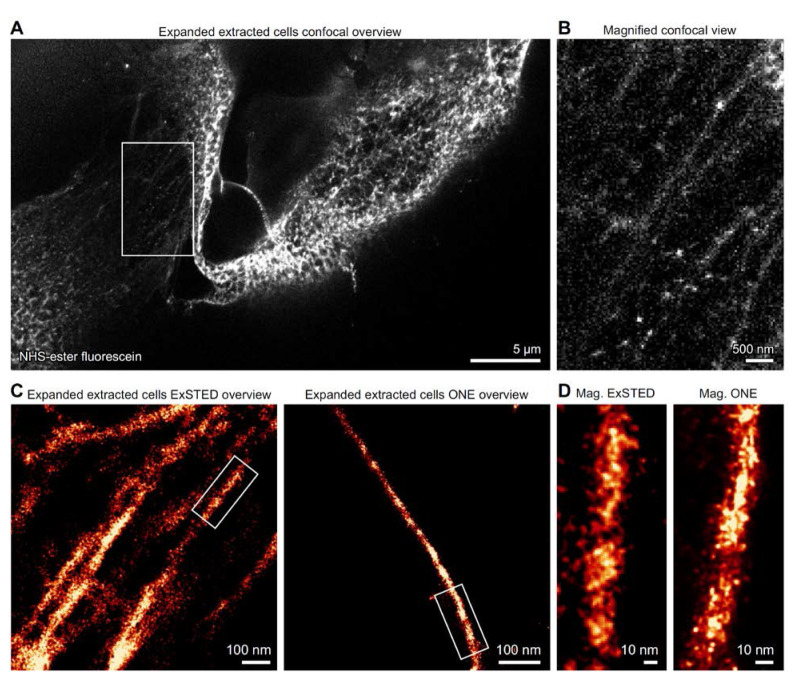
High resolution imaging of NHS-ester labeled extracted microtubules. (A) Confocal image of X10-expanded microtubules directly labeled with fluorescein NHS-ester. (B) A magnified region of the sample is shown for improved visualization. (C) Overviews of microtubules imaged using ExSTED (left) and ONE microscopy (right). (D) A magnified view of a single microtubule shows that comparable images can be obtained with these different imaging techniques; N = 2 independent experiments. Measurements and further validation of the imaged cytoskeletal elements are provided in [15].

For detailed imaging of expanded microtubules, we used a Leica TCS SP5 STED microscope in confocal mode. Samples were excited with either a 488 nm laser (for fluorescein) or a 633 nm laser (for STAR635P) through a HCX Plan Apochromat 100× 1.4 NA oil immersion STED objective. Emission signals were collected with HyD detectors and filtered by an AOTF at a scanning speed of 8 kHz. Images were acquired in 8-bit format at 128 × 128 pixels, with each pixel corresponding to 98 nm. Acquisition parameters in Leica LAS software were set to “minimize time interval,” and line averaging was avoided to preserve the intrinsic fluorophore fluctuations. Confocal images of expanded microtubules are shown in **Figure 4A, B**.

These confocal sequences were subsequently analyzed using the ONE plugin [18], available at (https://github.com/Rizzoli-Lab/ONE-Microscopy-Java-Plugin), yielding reconstructions beyond the diffraction limit. The resulting datasets are referred to as ONE images, after the one-step nanoscale expansion microscopy approach (**Figure 4C, D**).

STED imaging of expanded samples (ExSTED) provided higher apparent resolution compared to confocal acquisitions of expanded specimens [15]. Three-dimensional ExSTED imaging of extracted microtubules was performed on a Leica STELLARIS 8 microscope equipped with an HC PL APO 100×/1.4 oil-immersion STED White objective. Excitation was provided by a white light laser (WLL), tuned individually to the optimal wavelength for each fluorophore and operated at a pulse frequency of 80 MHz. Three-dimensional STED datasets were acquired with a theoretical pixel resolution of 20–40 nm. Depletion was achieved using a 775 nm pulsed STED beam at 80 MHz with an output power greater than 1.5 W, applied in combination with a 50 nm xy vortex donut and a 130 nm z donut. Fluorescence signals in the near-infrared and far-red ranges were collected using either Power HyD R SP or Power HyD X SP detectors, with a STED 3× notch filter in place. Detectors were operated in both photon-counting intensity mode and τSTED mode, depending on the experimental requirements.

## Data analysis

In detergent-extracted cells, cytoskeletal filaments remain preserved, with microtubules being the most prominent due to their larger diameter compared to thinner structures such as actin filaments. Open acquired image sequences in Fiji (ImageJ) for further processing, with specific plugins applied depending on the analysis goal.

*Note: Our detailed analysis procedure is provided in [15] and summarized briefly below.*

**ONE microscopy:** ONE images were generated from confocal acquisitions of expanded samples, as described in [15,18,25]. Image stacks were first aligned and corrected for drift before applying fluorescence fluctuation analysis. Local image correlations were computed frame-by-frame, yielding super-resolved reconstructions using the ONE plugin, a concept that adapts fluctuation analysis based on SOFI and SRRF [26,27]. For visualization, images were averaged after correlation analysis and filtered with a Gaussian kernel to suppress background noise while preserving filament continuity. The resulting datasets provided molecular-scale measurements of filament diameters and organization, which could then be quantified using line-profile analysis.

**ExSTED imaging:** ExSTED and deconvolved ExSTED (dExSTED) datasets were acquired as described in [15]. Both non-expanded and X10-expanded samples labeled with fluorescein NHS-ester were imaged on a STELLARIS 8 microscope (Leica Microsystems). Deconvolution was performed using the Lightning module in LAS-X software, which employs an accelerated, adaptive Lucy–Richardson algorithm (sometimes called lightning deconvolution). This algorithm incorporates regularization (Good’s roughness) and adapts to the local signal-to-noise ratio (SNR) per voxel, thereby reducing noise and avoiding over-amplification of background while preserving resolution. The gel–glass refractive index (RI) was measured empirically from four gels in four independent experiments using an Abbe-Refractometer AR4 (Krüss Optronic GmbH, Germany); this RI value of 1.33331 was then applied in the deconvolution settings. Image resolution was estimated by decorrelation analysis [28], with values calculated from 10 dExSTED images acquired from 10 different cells across 2 independent experiments, as shown in [15].

**Comparative measurements:** Filament dimensions obtained from dExSTED and ONE images were compared directly. Microtubules exhibited average widths of 24.98 ± 0.43 nm (28 cross-sections, N = 2 independent experiments), which is consistent with values reported from X-ray diffraction and cryo-EM studies [15]. In contrast, actin filaments were substantially smaller, with mean diameters of 7.41 ± 0.14 nm. These values were derived from 192 individual actin measurements across two independent experiments [18].

## General notes and troubleshooting

**General notes**

This protocol provides a robust method for extraction, gel integration, and subsequent labeling of microtubules for high-precision expansion microscopy. While optimized with specific reagents and conditions, the underlying principles are broadly applicable.

**Cell type compatibility:** The protocol was developed and validated in HeLa cells, but the detergent extraction and NHS-ester labeling principles are expected to work across other cell types and tissues. Extraction efficiency may vary, so minor optimization of detergent strength or incubation conditions may be required.

**Expansion protocol versatility:** We successfully utilized both X10 and ultrastructure expansion microscopy (U-ExM) protocols. In principle, the extracted microtubules obtained through this protocol are compatible with any other expansion gel recipe, including those for different expansion factors or specialized applications. For iterative ExM, we recommend performing NHS-ester labeling only after completion of the final expansion step. This minimizes potential interactions between gel polymerization chemistry and the dye, which could otherwise inactivate the fluorophore.

**NHS-ester dye selection:** NHS ester fluorescein and STAR635P were validated using this protocol, but other NHS-ester dyes should also be compatible, since they target primary amines on homogenized tubulin peptides. However, excessively hydrophobic dyes should be avoided, as they can adhere nonspecifically to negatively charged peptides, complicating dye removal and increasing background signal.

**Troubleshooting**

**Problem 1:** Less cell density in the expanded sample. Frequent washing steps in this protocol can lead to a progressive loss of cells, as they are not firmly attached to the coverslip via chemical coating. This reduction in cell density after extraction makes it challenging to locate suitable target cells in the expanded sample, which already exhibits a tenfold lower cell density.

Potential solution: When cells are plated on the evening before the experiment, the number should be adjusted in such a way that the confluency on the next morning reaches ~95%.

**Problem 2:** Poor anchoring due to harsh fixation. This protocol requires fixation of extracted cells using aldehyde. Poor fixation can result in breakdown of tubules during the expansion process. Fixed extracted cells are later anchored using Ac-X. Both fixative and anchoring molecules attack primary amines in the sample; hence, harsh fixation can potentially interfere with anchoring.

Potential solution: We observed that 0.5%–1% glutaraldehyde is optimal, which does not interfere with the anchoring process. We suggest not going beyond this limit.

**Problem 3:** Gel is not completely immersed in the dye solution. During post-expansion labeling, less volume of dye solution can only access the bottom side of the gel. It is difficult to predict which side of the gel the sample is anchored to. Hence, less accessibility of the dye solution on the top surface of the gel may lead to poor labeling of extracted microtubules.

Potential solution: Increase the volume of the dye solution in the labeling Petri dish so that it has access to both sides of the gel. This protocol uses a significantly higher concentration of the dye, and hence, dilution of the dye solution will not affect the quality of labeling.

**Problem 4:** Difficulty in finding the sample side of the gel. Usually, cells appear as surface irregularities in the X10 gel, which can be easily identified. However, in some cases, locating the sample side becomes tricky as those bumps are not that obvious.

Potential solution: Try to find the sample side of the gel using a microscope. Expected fluorescence should appear only from the sample side of the gel, while the other side should be dark.

**Problem 5:** Nanoscale translational motion of gel leading to substantial drift. During acquisition, the gel may drift, which can compromise image stability and alignment. Lateral (x/y) drift can arise from minor gel movements or thermal fluctuations, while vertical (z) drift is typically linked to microscope instability. This can arise from several factors:

1) Water accumulation: Water released from the gel can be collected between the coverslip and the support net, causing the gel to float instead of resting firmly on the coverslip.

2) Gel shrinkage: Gels are more than 90% water and begin to shrink as water evaporates, accelerated by heat from the excitation laser. This is typically the most severe cause of drift [29].

3) Gel misfit: If the gel slice is smaller than the support net, it may shift sideways during imaging.

4) Microscope instability: Using the microscope before it reaches thermal equilibrium can induce vertical (z) drift.

Potential solution:

1) Inspect the sample chamber for water accumulation on the coverslip. If present, carefully absorb with lens-cleaning paper to re-stabilize the gel.

2) If shrinkage has begun due to water loss, replace the gel slice with a fresh one. Using lower laser power can extend imaging time, allowing at least 1 h of acquisition before noticeable shrinkage occurs.

3) Avoid cutting the gel too narrowly; ensure slices fit tightly on the support net to reduce lateral movement.

4) Stabilize the microscope by leaving it powered on overnight to minimize z-drift.

*Note: The ONE analysis workflow can correct for moderate lateral (x/y) drift but not axial (z) motion, since z-slices are not incorporated into the alignment. Z-drift is usually not problematic because ONE images are acquired rapidly (<1 min), but ensuring that the microscope is thermally stable further reduces the risk of vertical displacement. For longer imaging regimes or alternative imaging modalities, drift correction plugins are available in Fiji [30,31].*

## Validation of protocol

This protocol has been used and validated in Chowdhury et al. [15] and Shaib et al. [18].

The extraction procedure was validated by observing the non-expanded, extracted cells with NHS-ester under the microscope. Objects closely resembling cytoskeletal elements were easily recognized, which validates the extraction procedure.

To validate accurate labeling after expansion, we compared fluorescence images of extracted microtubules in non-expanded cells (Figure 1) with those in expanded cells (Figure 4). The resemblance was obvious, indicating successful labeling. Furthermore, when we analyzed the expanded images at higher resolution using ONE microscopy [8], the filaments precisely matched the expected microtubule patterns. This observation was further supported by line scan analyses, which yielded average profiles of 24.98 nm [15]. This measurement aligns perfectly with previous data obtained from X-ray [16] and CryoEM [17] studies, providing strong evidence for the expansion factor accuracy of the expanded specimens.
